# Accuracy of anemia diagnosis by physical examination

**DOI:** 10.1590/S1516-31802007000300008

**Published:** 2007-05-03

**Authors:** Isabela Martins Benseñor, Ana Luísa Garcia Calich, André Russowsky Brunoni, Fábio Ferreira Espírito-Santo, Renato Lendimuth Mancini, Luciano Ferreira Drager, Paulo Andrade Lotufo

**Keywords:** Anemia, Diagnosis, Physical examination, Clinical competence, Hemoglobins, Anemia, Diagnóstico, Exame físico, Competência clínica, Hemoglobinas

## Abstract

**CONTEXT AND OBJECTIVES:**

Quantification of clinical signs such as the presence or absence of pallor at clinical examination is a key step for making diagnoses. The aim was, firstly, to evaluate two methods for anemia diagnosis by physical examination: four-level evaluation (crosses method: +/++/+++/++++) and estimated hemoglobin values, both performed by medical students and staff physicians; and secondly, to investigate whether there was any improvement in assessment accuracy according to the number of years in clinical practice.

**DESIGN AND SETTING:**

Forty-four randomly selected physicians and medical students in a tertiary care teaching hospital completed a physical examination on five patients with mild to severe anemia.

**METHODS:**

The observers used four-level evaluation and also predicted the hemoglobin level. Both methods were compared with the real hemoglobin value as the gold standard.

**RESULTS:**

The mean estimated hemoglobin value correlated better with the real hemoglobin values than did the four-level evaluation method, for attending physicians, residents and students (Spearman's correlation coefficients, respectively: 1.0, 1.0 and 0.9 for guessed hemoglobin and −0.8, −0.8 and −0.7 for the four-level evaluation method). There were no differences in the mean “guessed” hemoglobin values from attending physicians, residents and students. However, the correlation between guessed hemoglobin value and the four-level method was positive for attending physicians, thus suggesting some kind of improvement with time (p = 0.04).

**CONCLUSIONS:**

This study showed that estimated hemoglobin was more accurate than evaluation by the four-level method. The number of years in clinical practice did not improve the accuracy of clinical examination for anemia.

## RESUMO

**CONTEXTO E OBJETIVOS:**

A quantificação dos sinais do exame clínico como a presença de descoramento sugerindo anemia é um passo fundamental para o diagnóstico clínico. Avaliar dois métodos para o diagnóstico de anemia realizados por meio do exame físico-método de cruzes (+,++,+++,++++) e método de estimativa do valor de hemoglobina feito pelo médico ou estudante de medicina. Verificar se a acurácia desses métodos se relaciona com a experiência clínica.

**TIPO DE ESTUDO E LOCAL:**

44 médicos assistentes, residentes e estudantes de medicina selecionados de forma aleatória completaram o exame clínico de cinco pacientes com anemia leve a grave.

**MÉTODOS:**

Os examinadores utilizaram o método das cruzes em quatro níveis e, também estimaram o nível da hemoglobina. Ambos os métodos foram comparados com a hemoglobina medida (padrão-ouro).

**RESULTADOS:**

O valor estimado da hemoglobina se correlacionou melhor com a hemoglobina aferida por método automático do que a avaliação realizada pelo método das cruzes para as comparações realizadas entre médicos assistentes, residentes e alunos (coeficiente de correlação de Spearman respectivamente de 1,0, 1,0 e 0,9 para hemoglobina estimada e − 0,8, − 0,8 e − 0,7 para o método das cruzes). Não houve nenhuma diferença no valor estimado de hemoglobina entre os médicos assistentes, os residentes e os alunos. Entretanto, a correlação entre o valor estimado da hemoglobina e o método das cruzes somente foi positiva para os médicos assistentes, sugerindo algum tipo de melhora na avaliação da presença de anemia com a experiência clínica (p = 0,04).

**CONCLUSÕES:**

Este estudo mostrou que a estimativa do valor da hemoglobina pelo exame físico apresenta maior acurácia do que a avaliação pelo método de cruzes, considerando-se a medida automática de hemoglobina como padrão-ouro. A experiência clínica não melhora o diagnóstico de anemia.

## INTRODUCTION

Quantification of clinical signs such as the presence or absence of pallor at clinical examination is a key step for making diagnoses.^1^ A few studies in more recent years have tried to improve anemia diagnoses using pallor signs obtained from examining the hue of the conjunctivae, tongue, palms and nail bed. Although extreme clinical findings have great interobserver reliability, clinicians often disagree regarding the quantification of pallor in patients with mild anemia.^2–7^

Sheth et al. evaluated the value of conjunctival pallor for ruling in or ruling out the presence of severe anemia (hemoglobin < 9 g/dl) and to determine interobserver agreement. The likelihood ratios were 4.49 (95% confidence interval [CI]: 1.80-10.99) for the presence of conjunctival pallor, 1.80 (95% CI: 1.18-2.62) for borderline pallor and 0.61 (95% CI: 0.44-0.80) for the absence of conjunctival pallor. The interobserver agreement according to the kappa score ranged from 0.54 to 0.75 among paired observers. They concluded that the presence of conjunctival pallor was reason enough to perform a hemoglobin determination.^3^ In contrast, by evaluating conjunctival pallor for anemia diagnosis with a cut-off of 10 g/dl, Wurapa et al. found sensitivity of 18.6% and specificity of 95.8% and concluded that conjunctival pallor was not a good screening tool for anemia.^4^ This result has been confirmed by other studies.^5,6^

## OBJECTIVE

The present study addresses the accuracy of methods for diagnosing and quantifying anemia applied by medical students, residents and attending physicians and whether their abilities improve with experience.

## METHODS

A random sample of 10 attending physicians, 18 residents with less than five years of experience since graduation, and 16 fifth-year medical students working in Hospital das Clínicas, Faculdade de Medicina da Universidade de São Paulo (FMUSP), was invited to examine five in-patients on their first day of hospitalization. This study was approved by the Institutional Review Board and informed written consent was obtained from all participants. These patients had been admitted to hospital because of underlying diagnoses other than “anemia”. Hemoglobin levels were measured (using an automatic method) on the same day as the observations. We excluded patients who had previously been evaluated by these examiners elsewhere, and none of the participants had any prior information about any clinical data or laboratory test results.

The sample size was calculated for a category variable: presence or absence of anemia in an analytical study, with an effect size of 0.45, alpha (one-sided) of 0.05 and beta (two-sided) of 0.2. This totaled 13 individuals by group.^8^

The examiners were instructed to give an opinion after examining the hue of the conjunctivae, tongue, palms and nail bed. All physical examinations were performed at the bedside under artificial light. The examiners completed a questionnaire that asked them (1) to guess the likely hemoglobin value, and (2) which of the following methods were used to quantify anemia: a four-level evaluation (crosses method: +/++/+++/++++), a three-level evaluation (mild, moderate or severe), or a dichotomous evaluation (present or absent).

We created a score to compare the “guessed” hemoglobin with the real hemoglobin value that was measured as part of a complete blood cell count performed in an automated cell counter. The latter was taken to be the gold standard. The score formula was:


Score=1−[(estimated hemoglobin−real hemoglobin)/estimated hemoglobin]

Thus, if the estimated hemoglobin value was exactly the same as the real hemoglobin value, the numerator of the formula would be zero and the score equal to one.

We compared the mean “guessed” hemoglobin values, mean score values and mean number of crosses (four-level evaluation) by category of participants (medical students, residents with less than five years of clinical practice and physicians with more than five years of clinical practice) using analysis of variance (ANOVA) with the Bonferroni *post hoc* evaluation test for all examinations on each patient, and for all the patients together. For variables without normal distribution, non-parametric tests were used.

The “guessed” and real hemoglobin (gold standard) were only compared with the four-level evaluation method because 91% of the participants used this method to classify pallor. These comparisons were performed using Spearman's correlation test. The numbers of participants who used three-level or dichotomous evaluation were very small and this prevented other comparisons.

## RESULTS

We compared the four-level evaluation (crosses method) for diagnosing anemia with the real hemoglobin value obtained for each patient from a complete blood count (gold standard) that was performed using an automated cell counter, and, it was found that four patients presented mild to moderate anemia and one patient (patient 5) had severe anemia. Table 1 shows the characteristics of the five patients examined in the study, regarding age, gender, race, diagnosis and real hemoglobin value (gold standard).

**Table 1.** Baseline characteristics of the five patients in the study

Characteristics/patient	#1	#2	#3	#4	#5
Age (years)	72	48	21	50	22
Gender	Female	Female	Male	Male	Male
Skin color	Mixed	Mixed	White	Mixed	White
Final Diagnosis	Lymphoma	Myelodysplasia	Recovering from	Tuberculosis	Colon cancer
Hemoglobin level (g/dl)	9.0	9.8	10.1	12.6	7.1

Table 2 shows the mean estimated hemoglobin value, mean score value and mean number of crosses in the four-level evaluation, categorized into the three groups of examiners. There were no differences in the mean values for any patient between the groups.

**Table 2.** Analysis of variance and mean values (± standard deviation) of hemoglobin estimated by physicians, score and number of crosses, as methods for quantifying anemia, according to participant categories

	Students	Residents	Attending physicians	p
Mean estimated hemoglobin level (g/dl)				
Patient #1	8.6 ± 1.6	8.6 ± 1.6	9.5 ± 0.8	0.28
Patient #2	9.0 ± 1.0	8.8 ± 1.7	10.3 ± 1.5	0.24
Patient #3	10.7 ± 1.7	9.3 ± 3.2	10.1 ± 1.4	0.61
Patient #4	12.7 ± 0.8	11.7 ± 1.0	13.5 ± 2.2	0.06
Patient #5	8.1 ± 1.2	8.0 ± 0.7	8.7 ± 1.2	0.67
All patients	10.0 ± 2.2	9.4 ± 2.0	9.6 ± 1.6	0.43
Mean score level*				
Patient #1	0.82 ± 0.17	0.84 ± 0.17	0.94 ± 0.06	0.19
Patient #2	0.90 ± 0.12	0.78 ± 0.20	0.86 ± 0.06	0.31
Patient #3	0.86 ± 0.14	0.72 ± 0.23	0.89 ± 0.05	0.35
Patient #4	0.95 ± 0.03	0.90 ± 0.07	0.85 ± 0.01	0.11
Patient #5	0.89 ± 0.11	0.89 ± 0.08	0.83 ± 0.10	0.67
All patients	0.89 ± 0.10	0.83 ± 0.16	0.86 ± 0.10	0.43
Mean number of crosses (four-level)				
Patient #1	2.3 ± 0.7	2.7 ± 0.7	1.9 ± 0.4	0.054
Patient #2	2.4 ± 1.0	2.5 ± 0.9	1.7 ± 0.6	0.34
Patient #3	1.0 ± 0.6	2.0 ± 1.4	2.0 ± 0.001	0.14
Patient #4	1.0 ± 0.6	0.9 ± 0.3	0.5 ± 0.7	0.48
Patient #5	2.4 ± 0.5	2.5 ± 0.6	2.5 ± 0.7	0.90
All patients	1.9 ± 0.9	2.1 ± 1.0	1.9 ± 0.6	0.70

* *Score = 1 - [(estimated hemoglobin - real hemoglobin)/estimated hemoglobin].*

Spearman's correlation coefficient between the true hemoglobin value and the mean estimated hemoglobin level for each patient was 1.0 for both students and residents and 0.9 for the physicians. On the other hand, when the real hemoglobin level was correlated with the four-level evaluation, no significant association was observed for students (-0.8), residents (-0.8) or attending physicians (-0.7). Spearman's correlation coefficient between the four-level evaluation and the “guessed” hemoglobin was not significant for students (0.79) and residents (0.82), but it was statistically significant for attending physicians (0.90; p = 0.04).

Table 3 shows the sensitivity, specificity, positive and negative predictive values and positive and negative likelihood ratios of the estimated hemoglobin values for diagnosing mild anemia, using the total blood cell count as the gold standard. The estimated hemoglobin for diagnosing mild anemia had sensitivity of 64.6%, specificity of 83.6%, positive predictive value of 75%, negative predictive value of 92.4%, positive likelihood ratio of 3.9 and negative likelihood ratio of 0.4. In comparison, the four-level evaluation (crosses method) had slightly higher sensitivity of 78.6% but worse values for all other parameters: specificity (34.2%), positive predictive value (18.6%), negative predictive value (89.3%), positive likelihood ratio (1.2) and negative likelihood ratio (0.6).

**Table 3.** Sensitivity, specificity, positive and negative predictive values and positive and negative likelihood ratios for using hemoglobin estimated by physicians for diagnosing mild anemia, compared with total blood cell count as the gold standard

		Total blood cell count (gold standard)		Total
		Presence of mild anemia*	Absence of mild anemia	
**Estimated hemoglobin**	Presence of mild anemia	9	12	21
**(“guessing”)**	Absence of mild anemia	5	61	66
	**Total**	**14**	**73**	**87**

*Sensitivity = 64.3% (95%, confidence interval, CI, 35.6 to 86.0); specificity = 83.6% (95% CI, 72.7 to 90.9); positive predictive value = 42.9% (95% CI, 22.6 to 65.6); negative predictive value = 92.4% (95% CI, 82.5 to 97.2); positive likelihood ratio = 3.9 (95% CI, 2.0 to 7.5) and negative likelihood ratio = 0.4 (95% CI, 0.2 to 0.9).*

* *Considering hemoglobin 10.1-12 g/dl as mild anemia for women; and hemoglobin 11.1-13 g/dl as mild anemia for men; hemoglobin 8.1-10 g/dl as moderate anemia for women and hemoglobin 9.1- 11 g/dl as moderate anemia for men; and hemoglobin 8 g/dl for women and ≤ 9 g/dl for men as severe anemia.*

## DISCUSSION

The ability to guess a hemoglobin value following the physical examination was more accurate for diagnosing anemia or not than was the four-level evaluation method among patients with mild to severe anemia. No differences in assessment were found between medical students, residents and attending physicians, except for a positive correlation between the mean estimated hemoglobin value and the four-level evaluation, thereby suggesting some kind of improvement in the diagnosing ability over time. Compared with the four-level evaluation (crosses method), the estimated hemoglobin had higher specificity, positive and negative predictive values and better values for positive and negative likelihood ratios.

Previous data from another sample of medical students, residents and attending physicians showed that most of them used a four-level evaluation method to quantify anemia, as in the present study. Attending physicians with more than five years of clinical practice were more likely to make use of two-level evaluations than were residents or medical students.^1^ Considering that most of the physicians and physicians in training in the sample did not change their initial approach on the basis of the quantification of clinical signs (the initial management of a patient with an anemia of + or +++ would be the same), the usefulness of quantifying anemia using several categories (as in the case of the four-level evaluation method) needs to be questioned.

One important point is that although the accuracy of the four-level evaluation for anemia was worse than that of the estimated hemoglobin value for the same patient in this sample, in clinical practice and in the majority of medical schools the four-level evaluation is the most used and taught method for evaluating anemia. Another point is that the correlation between the four-level evaluation method and the real hemoglobin value did not increase with more years of clinical practice. In other words, an attending physician with more than five years of clinical practice is not more able to correctly evaluate the presence of anemia and its severity than is a physician in training. However, it is fair to say that some kind of improvement is still possible, because only for attending physicians was a positive correlation found between the four-level evaluation method and the mean “guessed” hemoglobin.

In the present study, we tried to choose patients with mild to moderate anemia because the difficulty in diagnosing or quantifying anemia is greater than in cases of severe anemia. However, even for the patient with severe anemia (patient #5), the correlation between the four-level evaluation method and the real hemoglobin value did not improve. The importance of diagnosing mild anemia is increasing, because anemia is now a valuable prognostic factor for many chronic disorders. In a sample of 100,000 hemodialysis patients, the presence of hematocrit higher than 30% was associated with a lower mortality rate. Subsequent treatment for anemia decreased the risk of death, after one year of follow-up.^9^ But if for patients with end-stage renal disease an anemia investigation is mandatory, it is not true for other chronic conditions. For example, patients with congestive heart failure are at higher risk of all-cause deaths, according to their lower hemoglobin levels. In a 15-month follow-up on 1,130 patients with low left ventricular ejection fraction, the participants who at enrollment were in the lowest quintile of hematocrit (25.4-37.5%) were found after multivariate adjustment to have a 52% higher risk of death than those in the highest quintile (46.1-58.8%).^10^ Other studies on patients with heart failure have confirmed these data.^11,12^

In the present cross-sectional study, all the examiners were under direct observation by the authors, in order to avoid differential misclassification. We decided to take into account a single evaluation by each examiner, instead of subcategorizing the physical examination according to the hue of the conjunctivae, skin, tongue and nail bed, as was done in other studies. Through this, we maximized the responses regarding the mean estimated hemoglobin value. Some patient characteristics such as skin color warrant investigation in the future.

## CONCLUSIONS

Evaluations of mild anemia are more effective using a “guessed” hemoglobin value than using the four-level evaluation method that is most commonly used and taught at medical schools. The data suggest a possible improvement in accuracy associated with greater numbers of years in clinical practice.
